# Syncope as the Initial Presentation of Severe Pulmonary Embolism Without Hypoxemia: A Clinical Case

**DOI:** 10.7759/cureus.87159

**Published:** 2025-07-02

**Authors:** Louis Wery

**Affiliations:** 1 Emergency Medicine, Cliniques Universitaires Saint-Luc, Bruxelles, BEL

**Keywords:** acute pulmonary embolism, emergency, emergency medicine resuscitation, high-risk pulmonary embolism, pulmonary embolism (pe), resuscitation, syncope, systemic thrombolysis, thrombolysis

## Abstract

Syncope and pulmonary embolism (PE) are common presentations in emergency medicine. While syncope is a recognized but rare manifestation of acute PE, it is exceptionally the sole initial symptom of PE. This case highlights the diagnostic and therapeutic challenges of such atypical presentations.

We present the case of a 71-year-old man admitted to the emergency department for isolated syncope. He was initially hypotensive, with no hypoxemia, chest pain, or dyspnea. Clinical examination and bedside echocardiography revealed signs of right ventricular (RV) dysfunction, leading to the diagnosis of high-risk pulmonary embolism. The diagnosis was confirmed by computed tomography pulmonary angiography (CTPA). The patient, with high-risk PE, underwent systemic thrombolysis. He required a brief hospitalization for monitoring in the intensive care unit (ICU) and has successfully recovered clinically.

This case illustrates the potential severity of PE presenting with isolated syncope and the value of focused cardiac ultrasound in emergency settings. Syncope in the context of PE is a major predictor of hemodynamic instability and early mortality. Despite its prognostic value, syncope is not currently integrated into risk stratification algorithms. The management of high-risk PE is guided by European Society of Cardiology (ESC) recommendations and includes systemic thrombolysis in the absence of contraindications. In this case, the patient required thrombolysis due to persistent hypotension.

Isolated syncope may be the only symptomatological manifestation of a life-threatening PE. Early recognition and risk-adapted management are critical to improving outcomes. This case highlights the importance of maintaining a high index of suspicion and utilizing point-of-care echocardiography in patients who present with syncope and hypotension.

## Introduction

Syncope and pulmonary embolism (PE) are common presentations in emergency medicine. While syncope is a recognized but uncommon manifestation of PE, its occurrence without accompanying dyspnea or hypoxemia is rare and diagnostically challenging. The prevalence of PE among patients presenting with syncope remains a matter of ongoing debate. Despite its low overall incidence, syncope in the context of acute PE is associated with more severe clinical presentations. A retrospective cohort study by Keller et al. showed that patients with PE who presented with syncope were more likely to be hemodynamically unstable [1]. The pathophysiological mechanisms underlying syncope in PE include abrupt reductions in cardiac output due to right ventricular (RV) overload, neurocardiogenic reflexes, and, less commonly, arrhythmias. These mechanisms highlight the central role of hemodynamic compromise in syncopal presentations. According to current European Society of Cardiology (ESC) guidelines [2], high-risk PE, characterized by cardiac arrest, obstructive shock, or sustained hypotension, requires urgent diagnosis and immediate reperfusion therapy, most commonly via systemic thrombolysis. Syncope, although not included in risk stratification, should raise clinical suspicion for high-risk PE, especially when associated with hypotension, shock, or signs of RV dysfunction.

This case report presents an atypical and severe PE revealed by syncope and hemodynamic shock, without respiratory symptoms or oxygen desaturation. Through this clinical scenario, we aim to emphasize the pathophysiological link between syncope and acute PE, review current guidelines for diagnosis and management of high-risk PE, and highlight the crucial role of bedside echocardiography and early reperfusion strategies in optimizing outcomes.

## Case presentation

A 71-year-old male patient was admitted to the emergency department via prehospital emergency medical service following two episodes of syncope. The first occurred during a Valsalva maneuver while straining on the toilet, and the second occurred while standing at the top of a staircase. No prodromal symptoms preceded either syncopal event.

Upon arrival at the emergency department, a systematic history did not reveal any associated complaints such as dyspnea, orthopnea, or paroxysmal nocturnal dyspnea. The patient also denied chest pain and palpitations.

His medical history included quadruple coronary artery bypass grafting (CABG), treated arterial hypertension, non-insulin-dependent type 2 diabetes mellitus, and obesity. He lived independently at home and maintained full autonomy in daily activities. The only venous thromboembolism (VTE) risk factor identified was a family history suggestive of thrombophilia, with no personal history of thromboembolic events.

At presentation, the patient's vital signs included hypotension (blood pressure: 86/45 mmHg), sinus tachycardia at 111 beats per minute, a respiratory rate of 18 breaths per minute, a peripheral oxygen saturation of 98% on ambient air, a body temperature of 36°C, and marked hyperglycemia at 21.1 mmol/L.

The physical examination was unremarkable except for clinical signs of circulatory shock: pallor, delayed capillary refill time (4 seconds), cold extremities, weak and thready radial pulses, and mottling of the lower limbs. Bilateral pitting edema was noted in the lower limbs and was reportedly stable per patient. No induration or tenderness along the venous pathways of the lower limbs was found; calves were supple, and Homans' sign was negative. Cardiopulmonary auscultation revealed tachycardia with no murmurs and no signs of elevated jugular venous pressure. Abdominal, genitourinary, and neurological examinations were noncontributory.

**Figure 1:**
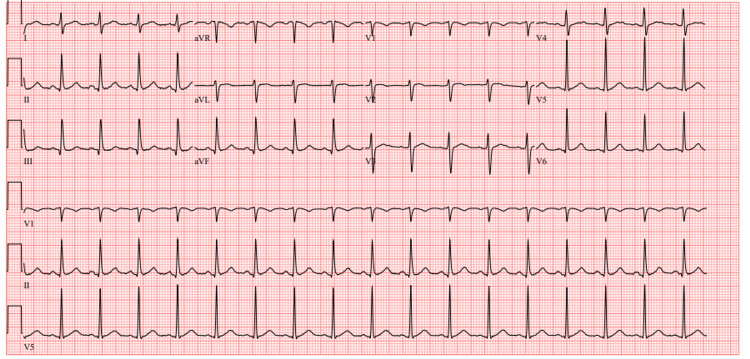
Electrocardiogram Sinus tachycardia (105 beats per minute)

An electrocardiogram revealed sinus tachycardia without associated repolarization abnormalities (Figure 1).

Given the clinical picture of circulatory shock, a fluid challenge was performed with 250 mL of crystalloid solution over 10 minutes. This resulted in a slight reduction in heart rate but no significant improvement in blood pressure.

Invasive arterial blood pressure monitoring via a radial artery catheter was performed. Arterial blood gas analysis of ambient air revealed metabolic acidosis with a pH of 7.33, partial pressure of carbon dioxide (PaCO_2_) of 27 mmHg, partial pressure of oxygen (PaO_2_) of 86 mmHg, lactate level of 6.33 mmol/L, bicarbonate level of 14 mmol/L, and base excess of -12 mmol/L (Table 1).

**Table 1:** Arterial blood gas PaCO_2_: partial pressure of carbon dioxide, PaO_2_: partial pressure of oxygen

Arterial blood gas	Patient value	Normal range
pH	7.33	7.35-7.45
PaCO_2_	27 mmHg	35-45 mmHg
PaO_2_	86 mmHg	75-100 mmHg
Lactate	6.33 mmol/L	0.5-1.5 mmol/L
Bicarbonate	14 mmol/L	22-28 mmol/L
Base excess	-12 mmol/L	-2 to 2 mmol/L

To investigate the etiology of the shock, bedside transthoracic echocardiography was performed, after crystalloid infusion, in the emergency department. It revealed impaired left ventricular (LV) systolic function with a moderately reduced ejection fraction estimated at 45%, paradoxical septal motion, a moderately dilated right atrium, and right ventricular dilation. There was no evidence of left ventricular hypertrophy or pericardial effusion.

These combined findings raised a high degree of clinical suspicion of PE presenting with obstructive shock. A bolus of unfractionated heparin (UFH) was administered. Following multidisciplinary discussion between emergency and intensive care physicians, a decision was made to proceed with computed tomography pulmonary angiography (CTPA) to confirm the diagnosis, given immediate scanner availability, followed by thrombolysis if indicated.

**Figure 2:**
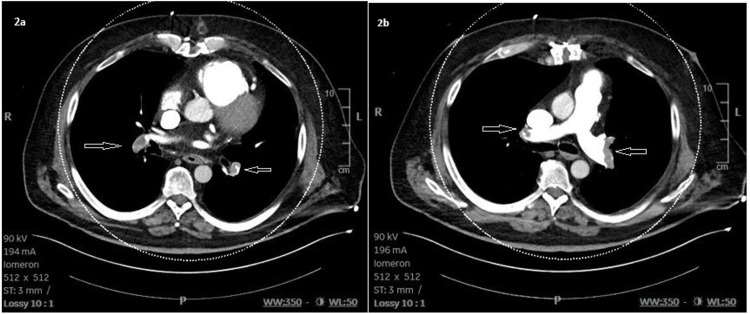
CTPA with central emboli Bilateral pulmonary embolism with intraluminal filling defects (yellow arrows). 2a: Thrombus is visible in the right and left interlobar pulmonary arteries. 2b: Thrombus is visible in both the right and left pulmonary arteries, consistent with a proximal pulmonary embolism. CTPA: computed tomography pulmonary angiography

CTPA confirmed massive bilateral pulmonary embolism with perfusion defects in both lung fields (Figure 2). CTPA was performed 30 minutes after admission and five minutes after bedside ultrasound.

Owing to persistent hypotension, absence of contraindications to thrombolysis, and a confirmed diagnosis of severe PE, systemic thrombolysis was initiated using alteplase (Actilyse®), with a 10 mg bolus, followed by a continuous infusion of 90 mg over two hours.

After thrombolysis, laboratory investigations revealed markedly elevated D-dimer levels (>7,650 ng/mL), increased high-sensitivity cardiac troponin T at 41 ng/L, normal N-terminal pro-B-type natriuretic peptide (NT-proBNP) at 855 pg/mL, and moderate renal impairment (creatinine of 1.9 mg/dL, urea of 99 mg/dL, and estimated glomerular filtration rate (eGFR) of 35 mL/minute/1.73 m^2^) (Table 2).

**Table 2:** Venous blood test NT-proBNP: N-terminal pro-B-type natriuretic peptide

Venous blood test	Patient value	Normal range
D-dimer	>7,650 ng/mL	<710 ng/mL
Troponin T (high sensitivity)	41 ng/L	<14 ng/L
NT-proBNP	855 pg/mL	<400 pg/mL
Creatinine	1.9 mg/dL	0.7-1.2 mg/dL
Urea	99 mg/dL	19-58 mg/dL

The patient was admitted to the intensive care unit (ICU) for monitoring under continuous UFH. Hemodynamically, he remained stable and did not require vasopressor or inotropic support during his ICU stay.

Further investigations revealed a deep vein thrombus extending from the proximal to middle segment of the right superficial femoral vein (part of the deep venous system) in the context of a heterozygous factor V Leiden mutation. Following a brief hospitalization in the pneumology department, the patient was discharged on apixaban therapy.

## Discussion

Epidemiology

Syncope is a common presentation, accounting for approximately 3% of emergency department visits [1] and 3% of hospital admissions via the emergency department [3]. It is defined as a transient loss of consciousness due to cerebral hypoperfusion, with a short duration and spontaneous and complete recovery [4]. Identifying the underlying etiology of syncope often represents a diagnostic challenge. It is important to emphasize that prevalence data on syncope should be interpreted with caution, given the heterogeneity in definitions and diagnostic approaches across studies evaluating syncope.

Pulmonary embolism is a frequent condition with an estimated global annual incidence of approximately one in 1,000 individuals [5]. It is the third leading cause of cardiovascular mortality, following myocardial infarction and stroke [2,6]. Mortality from PE in France ranges from 4.7% to 19.2% [7]. The most common cause is deep vein thrombosis [6].

The syncopal presentation of PE has long been recognized. As early as 1880, Dr. Luzzato described syncope as a clinical manifestation of acute PE [8]. Three main pathophysiological mechanisms have been proposed: (1) a vagally mediated Bezold-Jarisch reflex, (2) arrhythmia, and (3) decreased cerebral perfusion secondary to reduced cardiac output. Acute vascular occlusion leads to elevated pulmonary pressures, significantly increasing right ventricular (RV) afterload. The right ventricle, which is accustomed to low-pressure circulation, is unable to compensate, leading to reduced stroke volume and cardiac output [1,9,10].

Syncope can be an initial clinical manifestation of PE (with accompanying symptoms), with an estimated occurrence between 4% and 17% [6,11]. A retrospective study revealed that 9.8% of patients with PE presented with syncope [6]. However, in this study, syncope is never the sole clinical manifestation; it is commonly accompanied by hypoxemia, dyspnea, or, less frequently, electrocardiographic changes [6].

The actual prevalence of PE among patients presenting with syncope remains debated. In 2016, Prandoni et al. reported that one in six hospitalized patients with a first episode of syncope had an underlying PE on the basis of systematic screening [4]. Subsequent studies have not confirmed this prevalence. Most estimates suggest that less than 1%-1.5% of syncope cases seen in emergency departments are attributable to PE, with a prevalence of less than 3% among hospitalized patients [3,10,12,13]. Consequently, current guidelines do not recommend routine screening for PE in all cases of syncope [10,14]. The high prevalence reported by Prandoni et al. likely reflects systematic screening and the incidental detection of clinically silent PEs, including isolated subsegmental emboli [4].

The diagnostic approach to suspected PE is based on a pretest probability assessment using validated scores, such as the revised Geneva or Wells score. These tools guide further testing, including age-adjusted D-dimer assays and imaging, depending on the pretest probability. Empirical anticoagulation should be initiated without delay in patients with high or intermediate clinical probability [2].

Risk stratification and syncope

**Figure 3:**
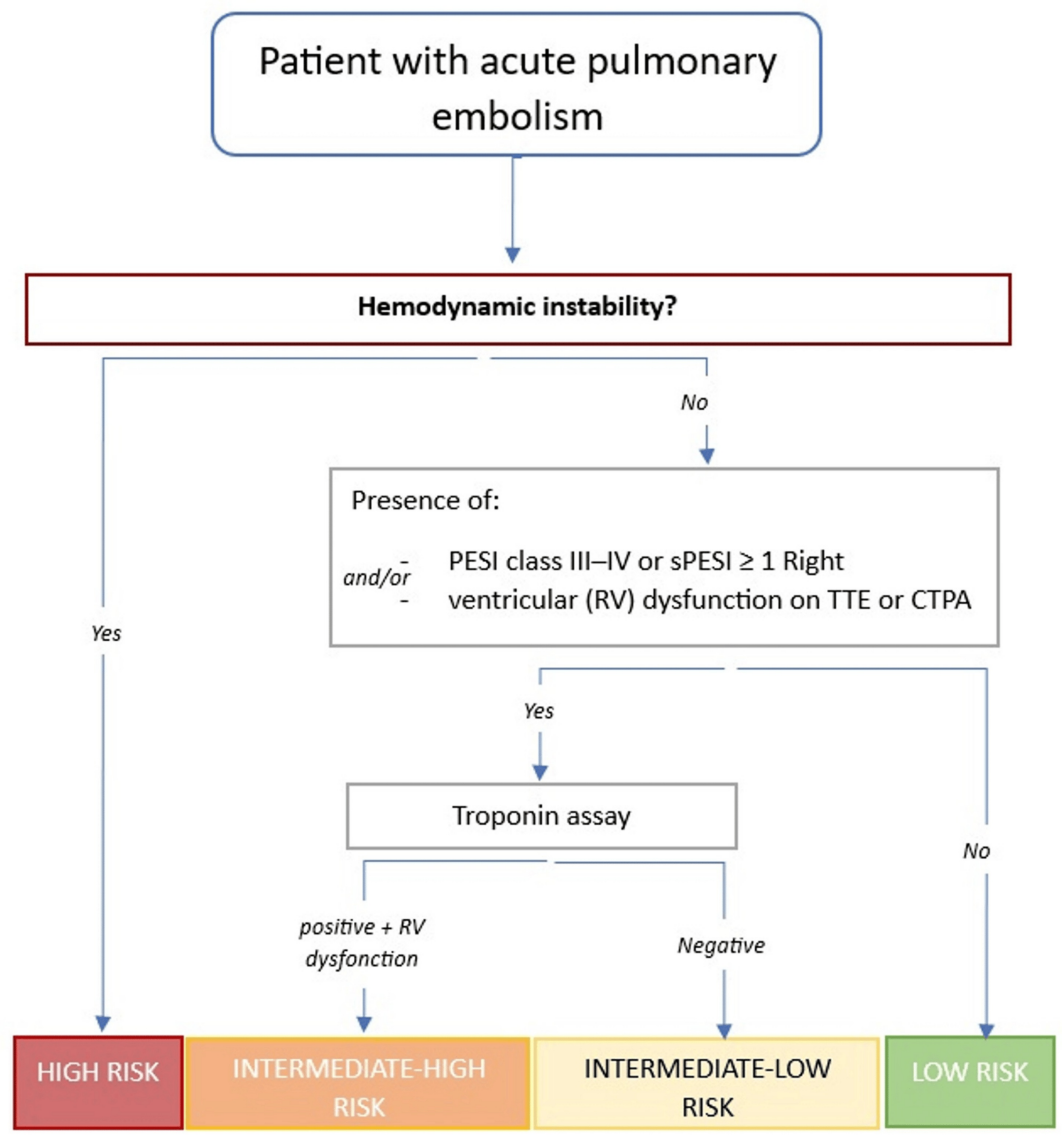
Risk stratification algorithm for acute pulmonary embolism Created and adapted from the ESC guidelines by Konstantinides SV, Meyer G, Becattini C, et al.: 2019 ESC Guidelines for the diagnosis and management of acute pulmonary embolism developed in collaboration with the European Respiratory Society (ERS): The Task Force for the diagnosis and management of acute pulmonary embolism of the European Society of Cardiology (ESC). Eur Respir J. 2019, 54:10.1183/13993003.01647-2019 [2] PESI: Pulmonary Embolism Severity Index, sPESI: simplified Pulmonary Embolism Severity Index, TTE: transthoracic echocardiography, CTPA: computed tomography pulmonary angiography, RV: right ventricle

Management decisions in PE rely heavily on risk stratification (class 1, level B) (Figure 3) [2], which incorporates hemodynamic stability, RV dysfunction, cardiac biomarkers, and the Pulmonary Embolism Severity Index (PESI).

High-risk PE is defined by hemodynamic instability: cardiac arrest, obstructive shock, or persistent hypotension. According to the ESC, persistent hypotension refers to a systolic blood pressure of <90 mmHg, a drop of ≥40 mmHg from baseline, or the need for vasopressors to maintain a systolic pressure of >90 mmHg for at least 15 minutes, in the absence of hypovolemia, sepsis, or arrhythmia [2]. High-risk PEs account for approximately 5% of PEs that present to the emergency department [2,7].

Due to persistent hypotension, our patient had, according to ESC stratification, a high-risk PE.

In suspected high-risk PE, immediate anticoagulation with UFH is recommended (grade 1C) [2], with a bolus of 80 IU/kg followed by a continuous infusion at 18 IU/kg/hour. Therapeutic monitoring should target either an activated partial thromboplastin time (aPTT) of 2-3× baseline or anti-Xa activity between 0.4 and 0.7 IU/mL, measured 5-6 hours after initiation. Dosage adjustments are made on the basis of these results. UFH is preferred due to its rapid onset, short half-life, and ease of titration, which are particularly relevant in unstable patients. It provides anticoagulation while allowing flexibility for urgent interventions such as thrombolysis or embolectomy.

The rationale for this risk stratification lies in the fact that hemodynamic instability is the main determinant of short-term mortality [2,15,16]. High-risk PE is associated with a mortality rate of 35%-38%, reaching 62.5% in ICU settings, and a three-month mortality of up to 52.3% [7].

Although not included in current risk stratification models, syncope has controversial prognostic significance. A systematic review and meta-analysis [17] revealed that patients with PE and syncope had significantly greater short-term mortality (18%) than those without syncope (8%), corresponding to an odds ratio (OR) of 1.95. These patients also had a higher prevalence of hemodynamic instability (OR: 4.87) and RV dysfunction [8,17]. However, in normotensive patients, the presence of syncope did not predict increased short-term mortality. Thus, the prognostic implication of syncope appears to be explained by its association with hemodynamic instability [17].

Imaging

In cases of suspected high-risk PE, bedside echocardiography is recommended to detect signs of RV dysfunction (grade 1C) [2]. Bedside ultrasound allows rapid assessment of a patient in shock to determine the underlying etiology. It provides a valuable time-saving tool and helps guide immediate therapeutic decisions. Key echocardiographic findings include RV dilation (RV/LV ratio), McConnell's sign, the D-shape of the left ventricle, the 60/60 sign, intracardiac thrombus, a reduced tricuspid annular plane systolic excursion (TAPSE < 16 mm), and a dilated, noncollapsible inferior vena cava. If the patient is stable and imaging is available, CTPA should be performed (class 1, level C). Otherwise, in the presence of RV dysfunction, treatment should proceed according to high-risk PE management protocols [2].

In our clinical case, bedside echocardiography revealed significant RV dilatation and paradoxical septal motion. These findings raised a high clinical suspicion of PE. Given the possibility of going immediately to the CTPA, it was decided to confirm the diagnosis before initiating thrombolysis.

Thrombolysis

According to current ESC guidelines, the severity of pulmonary embolism should be assessed to guide appropriate management strategies [2]. In cases of high-risk PE, as previously defined, numerous studies have demonstrated the beneficial effects of systemic thrombolysis in combination with UFH for rapid reperfusion. Therefore, in the absence of contraindications (Table 3), systemic thrombolysis is the recommended first-line treatment (class 1, level B) for high-risk patients.

**Table 3:** Contraindications to thrombolysis Created and adapted from the ESC guidelines by Konstantinides SV, Meyer G, Becattini C, et al.: 2019 ESC Guidelines for the diagnosis and management of acute pulmonary embolism developed in collaboration with the European Respiratory Society (ERS): The Task Force for the diagnosis and management of acute pulmonary embolism of the European Society of Cardiology (ESC). Eur Respir J. 2019, 54:10.1183/13993003.01647-2019 [2] TIA: transient ischemic attack

Absolute contraindications to thrombolysis	Relative contraindications to thrombolysis
History of hemorrhagic stroke or stroke of unknown origin	TIA within the past 6 months
Ischemic stroke within the past 6 months	Current oral anticoagulation
Central nervous system neoplasm	Uncontrolled hypertension
Major trauma, surgery, or head injury within the past 3 weeks	Active peptic ulcer disease
Hemorrhagic diathesis	Severe hepatic failure
Active bleeding (excluding menorrhagia)	Pregnancy or first week postpartum
	Known bleeding diathesis
	Recent noncompressible vascular puncture

Based on the ESC stratification, our patient had a high-risk PE and, according to guidelines, had to be treated by thrombolysis even if CTPA was not performed. Although systemic thrombolysis was promptly administered due to the patient's unstable condition, it is important to briefly consider alternative reperfusion strategies such as catheter-directed thrombolysis (CDT) or surgical embolectomy, particularly in cases with contraindications to systemic lysis or failure to respond. In case of persistent hemodynamic instability despite thrombolysis, extracorporeal membrane oxygenation (ECMO) should be considered as a bridge to these alternative reperfusion strategies.

The Pulmonary Embolism Thrombolysis Trial revealed that thrombolytic therapy reduces the risk of cardiopulmonary collapse, but it is associated with a significantly increased risk of intracranial and other major bleeding events [18,19]. Given the high mortality associated with hemodynamic instability, the benefits of thrombolysis outweigh the hemorrhagic risks in this high-risk population.

While systemic thrombolysis has been shown to reduce early mortality among patients in shock, evidence does not support a long-term survival benefit in patients with intermediate-high risk PE [7]. There was a significant increase in the risk of major bleeding associated with thrombolysis. Notably, the risk of intracranial hemorrhage was 10 times higher in the thrombolysis group, occurring in 2% of patients compared to only 0.2% in the placebo group. In addition, other major bleeding events were reported in 11.5% of patients treated with thrombolysis versus 2.4% in the placebo group [19]. A meta-analysis further demonstrated that although thrombolysis reduces PE-related mortality in this subgroup, it does not decrease all-cause mortality [7]. As a result, patients with intermediate-high-risk PE should be closely monitored, and thrombolytic therapy should be considered only in cases of clinical deterioration or progression to hemodynamic instability (class 1, level B) [2,15].

Approximately 5% of patients with intermediate-high-risk PE experience clinical worsening, usually within an average of 1.8 days after presentation, and may ultimately require rescue thrombolysis [7].

Compared with UFH alone, systemic thrombolysis facilitates more rapid relief of pulmonary arterial obstruction [2,7]. This leads to faster reductions in pulmonary arterial pressure, decreased right ventricular dilatation, improved perfusion defects, and enhanced arterial oxygenation [18].

The maximum therapeutic benefit of thrombolysis is observed when it is administered within the first 48 hours of symptom onset [2]. However, treatment can still be considered up to 6-14 days after initial symptom presentation in selected cases [2].

Thrombolysis failure is defined as persistent hemodynamic instability and right ventricular dysfunction 36 hours after thrombolytic administration [2].

When systemic thrombolysis is indicated, recombinant tissue plasminogen activator (rtPA) (alteplase) is the recommended agent, administered as a 100 mg infusion over two hours following a 10 mg initial intravenous bolus (Table 4) [2].

**Table 4:** Alteplase administration Created and adapted from the ESC guidelines by Konstantinides SV, Meyer G, Becattini C, et al.: 2019 ESC Guidelines for the diagnosis and management of acute pulmonary embolism developed in collaboration with the European Respiratory Society (ERS): The Task Force for the diagnosis and management of acute pulmonary embolism of the European Society of Cardiology (ESC). Eur Respir J. 2019, 54:10.1183/13993003.01647-2019 [2] mg: milligrams, h: hour, kg: kilograms, rtPA: recombinant tissue plasminogen activator

	Administration mode	Alteplase dose
Alteplase (rtPA)	Bolus dose	10 mg
100 mg over 2 hours	Continuous infusion	90 mg over 2 hours (1.5 mg/kg, max 90 mg)

Coadministration of UFH with alteplase is also recommended, as all major clinical trials evaluating thrombolytic therapy for PE have used this combination regimen [2].

Other therapeutic options

Surgical embolectomy is recommended for patients with contraindications to systemic thrombolysis (class 1, level C) (Table 3) [6,14], patients with failed thrombolysis (class 1, level C) [7], patients with intracardiac thrombi, or pregnant patients [20]. This intervention requires cardiopulmonary bypass, thus necessitating management in specialized centers with experienced cardiothoracic surgeons.

Although no randomized controlled trials have directly compared surgical embolectomy versus systemic thrombolysis, observational data suggest similar mortality outcomes between the two strategies [7,20]. However, this lack of difference may be influenced by selection bias, as surgical embolectomy is often reserved for more critically ill or refractory patients. The overall mortality of surgical embolectomy is approximately 12%, increasing to 32% in the context of cardiac arrest [19].

Catheter-directed thrombolysis (CDT) has emerged as a minimally invasive alternative to surgical embolectomy (class 2a, level C) [20], provided that the necessary expertise and resources are available. This field is evolving rapidly, with several ongoing clinical trials. However, the current evidence is limited to small randomized studies and observational series, resulting in scarce and heterogeneous data.

The reported clinical success rate of CDT is approximately 87%, with success defined as patient survival, hemodynamic stabilization, and improvement in oxygenation. As more robust comparative data become available, catheter-directed therapies may reshape current practices, especially if they are shown to match or surpass systemic thrombolysis in terms of safety and efficacy.

In cases of refractory circulatory collapse or cardiac arrest, ECMO may be considered adjunctive support in combination with surgical embolectomy or catheter-directed thrombolysis (class 2b, level C) [2]. ECMO provides right ventricular unloading and adequate peripheral perfusion, acting not as a definitive therapy, but as a bridge to intervention in patients with high-risk PE and profound hemodynamic compromise [7]. As with surgical options, this approach requires advanced infrastructure and multidisciplinary expertise.

In parallel with definitive reperfusion therapies, shock management must be promptly initiated. This includes cautious fluid resuscitation (taking into account ventricular interdependence and Starling's law) and the administration of inotropes (e.g., dobutamine) and vasopressors (e.g., norepinephrine) (class 2a, level C) [7]. These medications were not required in our patient but remain important in management if indicated. Invasive mechanical ventilation should be avoided whenever possible, as it may further compromise hemodynamics in these critically ill patients.

Practical recommendations

Patients presenting with syncope due to pulmonary embolism (PE) should be closely monitored, given the high risk of hemodynamic instability. In the presence of hemodynamic instability, systemic thrombolysis remains the recommended first-line treatment, provided that there are no contraindications. Point-of-care ultrasound (POCUS) plays a critical role in the rapid diagnostic assessment of patients in shock and may guide immediate therapeutic decisions. In cases of suspected high-risk PE, anticoagulation should be initiated empirically while awaiting imaging confirmation, if the bleeding risk is acceptable.

Further comparative studies evaluating interventional techniques (e.g., catheter-directed therapies) versus systemic thrombolysis may lead to changes in clinical practice.

## Conclusions

This clinical case, centered on a common complaint (syncope), illustrates its potential to reveal a life-threatening but frequently encountered diagnosis: acute pulmonary embolism (PE). Although this association has been recognized for years, syncope as the sole symptomatological manifestation of PE remains rare, which was the case for our patient. Syncope is a major predictor of hemodynamic instability in the context of PE, and such instability is the primary determinant of early mortality. Despite its clinical relevance, current guidelines do not recommend systematic screening for PE at all syncopal presentations because of the low overall prevalence of PE among patients with syncope. This case underscores the potential need to re-evaluate the role of syncope in PE risk models and emphasizes the importance of considering PE in cases of unexplained syncope accompanied by shock or hypotension.

The therapeutic management of PE is guided by risk stratification, in which syncope is not currently included. POCUS plays a key role in early risk assessment and the rapid identification of RV dysfunction and should be integrated into emergency evaluations of patients presenting with unexplained syncope accompanied by shock or hypotension. For high-risk PE, which is defined by cardiac arrest, obstructive shock, or persistent hypotension, systemic thrombolysis is the first-line treatment in the absence of contraindications (class 1, level B). In the presence of contraindications or failed thrombolysis, surgical or catheter-based thrombectomy is indicated, while extracorporeal membrane oxygenation (ECMO) serves as a hemodynamic support measure in refractory shock, but it must be used in conjunction with a definitive reperfusion therapy.

## Figures and Tables

**Figure 4:**
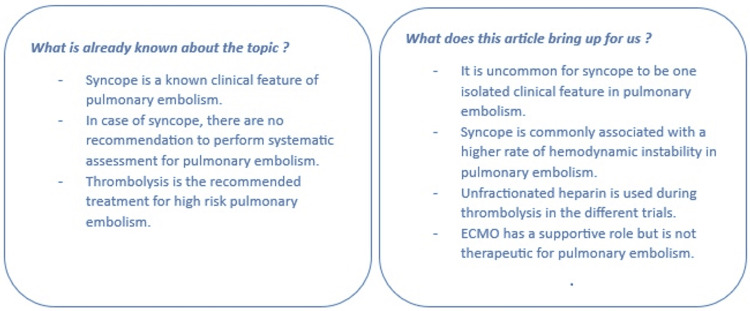
What is already known about the topic and what does this article bring up for us? Summary created by the author ECMO: extracorporeal membrane oxygenation

## Appendices

Figure 4 provides a summary of the existing knowledge on the topic and the new insights presented in this article.
